# The evolution of genital complexity and mating rates in sexually size dimorphic spiders

**DOI:** 10.1186/s12862-016-0821-y

**Published:** 2016-11-09

**Authors:** Matjaž Kuntner, Ren-Chung Cheng, Simona Kralj-Fišer, Chen-Pan Liao, Jutta M. Schneider, Mark A. Elgar

**Affiliations:** 1 Institute of Biology, Research Centre of the Slovenian Academy of Sciences and Arts, Ljubljana, Slovenia; 2 National Museum of Natural History, Smithsonian Institution, Washington, DC USA; 3 Department of Life Science, Tunghai University, Taichung, Taiwan; 4 Zoological Institute, Biozentrum Grindel, University of Hamburg, Hamburg, Germany; 5 School of BioSciences, University of Melbourne, Victoria, 3010 Australia

**Keywords:** Sexual selection, Sexual size dimorphism, Sexual conflict, Female gigantism, Sexually antagonistic coevolution, *Nephila*

## Abstract

**Background:**

Genital diversity may arise through sexual conflict over polyandry, where male genital features function to manipulate female mating frequency against her interest. Correlated genital evolution across animal groups is consistent with this view, but a link between genital complexity and mating rates remains to be established. In sexually size dimorphic spiders, golden orbweaving spiders (Nephilidae) males mutilate their genitals to form genital plugs, but these plugs do not always prevent female polyandry. In a comparative framework, we test whether male and female genital complexity coevolve, and how these morphologies, as well as sexual cannibalism, relate to the evolution of mating systems.

**Results:**

Using a combination of comparative tests, we show that male genital complexity negatively correlates with female mating rates, and that levels of sexual cannibalism negatively correlate with male mating rates. We also confirm a positive correlation between male and female genital complexity. The macroevolutionary trajectory is consistent with a repeated evolution from polyandry to monandry coinciding with the evolution towards more complex male genitals.

**Conclusions:**

These results are consistent with the predictions from sexual conflict theory, although sexual conflict may not be the only mechanism responsible for the evolution of genital complexity and mating systems. Nevertheless, our comparative evidence suggests that in golden orbweavers, male genital complexity limits female mating rates, and sexual cannibalism by females coincides with monogyny.

**Electronic supplementary material:**

The online version of this article (doi:10.1186/s12862-016-0821-y) contains supplementary material, which is available to authorized users.

## Background

Sexual conflict over mating frequency [1] may create a sexually antagonistic selective regime, thought to be responsible for the coevolution of male and female traits that facilitate protection of evolutionary interests within each sex, and at the same time limit the mating frequencies of the other sex [2–4]. Sexually antagonistic co-evolutionary stages are characterized by the interaction between sets of male persistence traits and female resistance counter-adaptations [5]. A classic example of sexual conflict is when male traits that protect male paternity by inhibiting polyandry subsequently act as selection pressures favoring counter-acting female traits that prevent male monopolization [5, 6]. Male persistence traits may include harmful genitalia [7], accessory gland products [8], and genital mutilation and plugging [9]. Female counter-adaptations may include modifications of female genital anatomy [10], physiological adjustments [11], and concealment of paternity [12]. Females may also engage in pre- or post-copulatory sexual cannibalism, thereby preventing unwanted copulations [13–16]. The intensity of sexual conflict and thus strength of selection acting on these traits may be influenced by the potential mating rates of both males and females [2, 17].

Sexual conflict is an ongoing process, the intensity of which varies between populations and species, and may drive diversification, speciation, and extinction rates [17]. The nature of sexual conflict and its role in phenotypic evolution remain elusive [18, 19], and may be either a cause or consequence of evolving traits. Consequently, phylogenetic comparative studies are a useful approach to elucidating the role of sexual conflict at macroevolutionary scales [5].

Animal genitalia are diverse and evolve relatively rapidly compared with somatic traits [20–23]. The extraordinary diversity of male and female genitalia may partially derive from sexual conflict over mating rates, where particular features of the genitalia of one sex function to manipulate mating frequency against the interest of the other sex [17]. The correlated evolution of male and female genitalia, revealed by comparative analyses [7, 10, 22, 24–27] may be consistent with the predictions of sexual conflict over mating frequency, which also requires sexual selection as its component [28]. Critically, the nature of the sexual conflict is not revealed by the majority of these studies [18, 19], which do not explore how the evolutionary trajectory of genital traits, such as complexity, is linked to the mating rates of males, females or both.

Studies that integrate these aspects have measured female mating rates and the intensity of sexual conflict in a clade of water striders [17, 22, 29]. However, the evolutionary role of sexual conflict beyond water striders remains poorly understood, and this is particularly true for spiders, a mega-diverse invertebrate order with impressive variation in somatic and genital morphology, and extreme sexual repertoires [14, 18, 30–33]. Golden orbweaving spiders (family Nephilidae) are extremely sexually size dimorphic with females on average up to 125 times heavier than their mates [34]. The evolution of body size in nephilids is decoupled between the sexes [35, 36]. The resulting extreme female biased sexual size dimorphism introduces issues of genital size mismatches between males and females [37], and as a consequence, components of male and female genitalia may evolve at differing rates to compensate for such mismatches [38].

The suggested evolutionary link between male genital complexity and its impact on female mating rates has not been tested in a phylogenetic framework. Relatively small nephilid males of certain species engage in extreme mating strategies, including severing terminal parts of their pedipalps (sperm transferring appendages), which are used to plug female copulatory openings [39, 40]. Experimental studies on selected species found that plugs from males with complex genitals commonly prevent female polyandry, whereas plugs from simple genitals do not [39, 41]. Assuming that male strategies to monopolize paternity with a single female via genital plugging are not in the interest of the female [20], females ought to evolve counter-adaptations. These could be behavioral and might include aggression and sexual cannibalism [14, 33], or might involve morphological adjustments in genital morphology [26].

Using all nephilid species from a recent phylogeny [42], we retest the pattern of genital complexity coevolution between the sexes in nephilid spiders [26, 43], then examine phylogenetic associations between mating rates and male morphological and female behavioral traits. Specifically, we predict a negative correlation between female mating rates and male genital complexity, if more elaborate male palps function to prevent female polyandry. We also predict a negative correlation between male mating rates and sexual cannibalism if post-copulatory cannibalism functions as a female mechanism of preventing male-imposed monandry.

## Methods

### Genital complexity scores

Genital complexity scores (Additional file 1: Table S1) were obtained from a prior study [26] that used 10 genital features per sex as counts of summed complexity. Briefly, this approach scores the presence of male features such as sclerite ridges, flaps and hooks (Additional file 2: Figure S1, Additional file 3: File S1), that contribute to overall palpal complexity, and female features such as hooks and duct curls that contribute to complexity of external (epigynal) and internal (vulval) genital anatomy [26]. We modified this dataset for a more precise taxonomic match with the new phylogeny, thus adding data for *Herennia oz* scored from the genus revision [44], for both sexes of *Clitaetra thisbe* updated from two *Clitaetra* taxonomic treatments [45, 46], and with updated *Nephilingis* taxonomy [47]. We left the outgroup *Zygiella* unscored as it is unclear whether or not this taxon possesses the embolic conductor shared by nephilids and the group *Deliochus* + *Phonognatha* [43], a morphological feature central to nephilid genital complexity scoring.

### Genital damage and mating rates

In nephilid spiders, males break off distal parts of their pedipalps to form genital plugs, but these plugs, lodged in female copulatory openings, do not necessarily prevent female polyandry. Our morphological examinations on the prevalence of genital plugs, consisting of palpal parts from a single versus multiple males [43, 44], helped score for male genital damage presence or absence for most taxa in the phylogeny (Additional file 1: Table S1). Additional evidence comes from detailed species level experimental studies [39, 41, 48–53].

We simplified the male and female mating rates to a dichotomy that reflects monogamy (monandry or monogyny) versus polygamy (polyandry or polygyny). We define polygyny as male mating with more than one female, whereas monogynous males invest into repeated mating with the same female in an attempt to plug both of her copulatory openings. We based the inferred mating rates in nephilids and outgroups (Additional file 1: Table S1) on available experimental studies [13, 39–41, 48–73] and on genital damage data where single versus multiple male mating plugs per female copulatory opening predict monandry and polyandry, respectively [26, 35, 40, 41, 53]. Most *Nephila* species, and *Phonognatha graeffei,* are polyandrous [49, 61]. Based on experimental studies, we deemed a male-enforced monogamy in *Herennia*, *Nephilengys* and *Nephilingis* [74]. While little is known about the sexual biology of *Clitaetra*, their genitals are never plugged, hinting at possible polyandry. Our inferred mating rate scores match the established mating systems in those cases where experimental data are available (Additional file 1: Table S1).

### Body length, SSD, and sexual cannibalism

We used sexual size dimorphism (SSD) indices [36] as ratios of mean female body length to male body length (Additional file 1: Table S1). Because sexual cannibalism strongly depends on the mating status of the female, we translated the experimental data on post-copulatory sexual cannibalism by virgin females [13, 39, 48, 49, 51, 56, 60–62, 64, 72, 75–77] to average percentage scores per species (Additional file 1: Table S1).

### Phylogeny

The coevolutionary pattern of nephilid male and female genital complexity [26] relied on a phylogeny that lacked branch lengths (Additional file 4: Figure S2A; [43]). The reference nephilid phylogeny used here was recently proposed through rigorous analyses of 4 k bp nucleotide data obtained for 28 out of 40 nephilid species and numerous outgroups (Additional file 4: Figure S2B; [42]). We pruned the reference phylogeny for any redundant ingroup taxa and for most outgroup taxa retaining only the immediate sister clade to nephilids. The resulting base phylogeny had 30 terminals (Additional file 1: Table S1). Note that all comparative analyses are based on the same reference topology, but adjust terminal numbers to avoid missing taxon scores that would preclude specific comparative testing (see below).

### Comparative analyses

We tested for correlations between pairs of continuous variables (genital complexity, body size, SSD, sexual cannibalism) using phylogenetically independent contrasts (PIC) analysis [78] in the PDAP module of Mesquite 3.0 [79]. All continuous variables passed the PDAP test for data conformity, thus we used the inferred, untransformed branch lengths in combination with two tailed *P* values.

We explored the relationships between continuous (male and female genital complexity, male and female body length, SSD, cannibalism rate; Additional file 1: Table S1) and discrete (inferred male and female mating rates; Additional file 1: Table S1) traits using three different analyses. We explored associations between male and female mating rates on the one hand and each continuous trait on the other using phylogenetic ANOVA [80] implemented as function ‘phylANOVA’ in the R package ‘phytools’ [81], and generalized estimating equations (GEE) [82] implemented via function ‘compar.gee’ with default settings in the R package ‘ape’ [83]. We then ran multiple variable regression analyses using a Bayesian generalized linear mixed model (GLMM) with a logit link function within the R package ‘MCMCglmm’ [84]. This approach takes into account phylogenetic relationships by using phylogeny as a covariate [85] and analyzes continuous traits as independent variables simultaneously to test their association with the dependent factor, in our case male and female mating rates. To avoid the collinearity in the GLMM analysis, we first conducted an exploratory factor analysis of the five independent variables with direct oblimin rotation using ‘fa’ function in R package ‘psych’ [86]. Exploratory factor analysis revealed three independent factors that were used in subsequent GLMM analyses (Additional file 5: File S2): MR1 related to SSD and female body length, MR2 related to male and female body length, and MR3 related to male and female genital complexity.

## Results

**Fig. 1:**
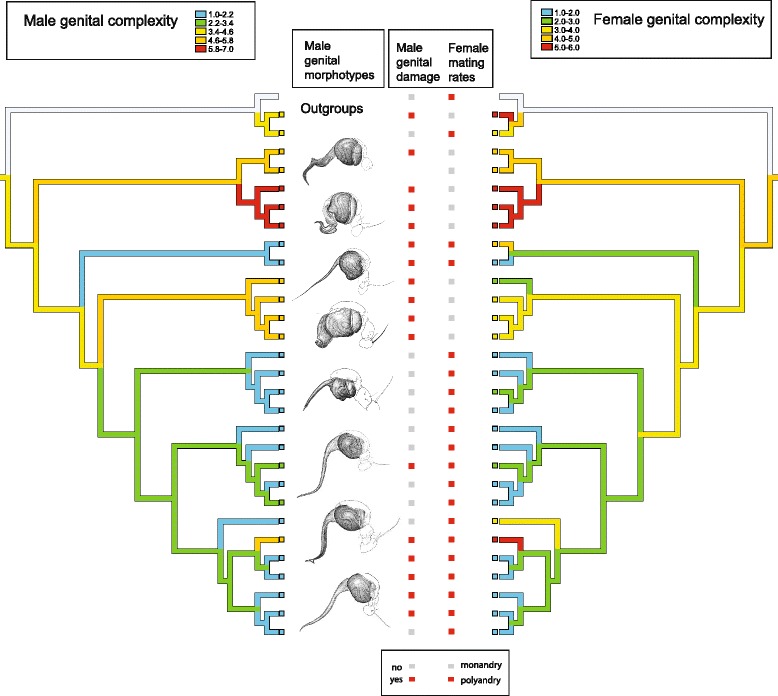
Summarized trait optimization in nephilid spiders. Ancestral states are reconstructed using parsimony optimization on a Bayesian phylogeny. Terminal names and branch length information are omitted for clarity; instead, typical male palpal anatomies are shown, and simple scores for male genital damage and female mating rates are given. Male and female genital complexity show positive phylogenetic correlation (PIC, *P* < 0.0001)

PIC analyses reveal a significant positive correlation between male and female genital complexity (*R* ^*2*^ = 0.437, *t* = 4.851, *d.f.* = 27, *P* < 0.001; Fig. 1), confirming the prior pattern of concerted male and female genital evolution [26]. Neither female nor male genital complexity showed any phylogenetic correlation with sexual size dimorphism (SSD) or with male body size (Female genital complexity vs. SSD: *R* ^*2*^ = 0.012, *P* = 0.586; Male genital complexity vs. SSD: *R* ^*2*^ = 0.010, *P* = 0.612; Female genital complexity vs. male body size: *R* ^*2*^ = 0.024, *P* = 0.427; Male genital complexity vs. male body size: *R* ^*2*^ = 0.051, *P* = 0.248). However, in species with larger females, male and female genitals were simpler (Female genital complexity vs. Female body size: *R* ^*2*^ = 0.163, *P* = 0.029; Male genital complexity vs. Female body size: *R* ^*2*^ = 0.153, *P* = 0.035).

**Fig. 2:**
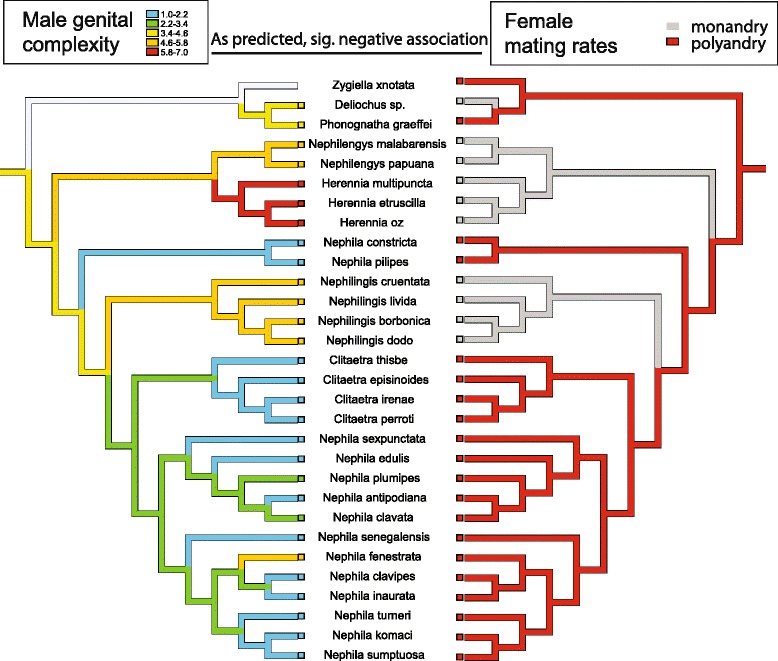
Reconstructed evolution of male genital complexity and female mating rates. Comparative analyses suggest that these variables are negatively correlated (phylogenetic ANOVA, *P* = 0.002; GEE, *P* < 0.001)

Phylogenetic reconstructions (Figs. 1 and 2) suggest that the evolution of male genital complexity is negatively associated with female mating rates: the evolutionary maintenance of polyandry-reconstructed as an ancestral trait-coincides with repeated shifts to simplified genital anatomy, while two independent origins of monandry in Nephilidae (though not in the outgroup *Deliochus*) coincide with shifts to increased male genital complexity.

Consistent with our first prediction, phylogenetic ANOVA and GEE analyses (Table 1, Additional file 6: Figure S3) reinforce this pattern by establishing a significant negative association between female mating rates and male genital complexity (with polyandry being more likely in species with simpler male genitals). These analyses also suggest that male mating rates are negatively associated with male and female genital complexity (with polygyny being more likely in species with simpler male and female genitals).

**Table 1:** The results of phylogenetic ANOVA and generalized estimating equations (GEE) analyses testing for association between discrete traits and continuous characters

	Phylogenetic ANOVA		GEE	
	*F* value	*P* value	Slope (±SE)	*P* value
Female mating rate				
vs. female genital complexity	16.494	0.106	−0.191 (1.019)	0.096
**vs. male genital complexity**	**75.296**	**0.002**	**−3.747 (0.692)**	**<0.001**
vs. female body length	1.951	0.550	1.811 (7.611)	0.818
vs. male body length	1.079	0.656	0.099 (1.307)	0.941
vs. sexual size dimorphism	0.370	0.797	0.121 (1.630)	0.942
vs. sexual cannibalism rate	4.750	0.219	−0.242 (0.169)	0.224
Male mating rate				
**vs. female genital complexity**	**25.112**	**0.002**	**−1.851 (0.417)**	**0.002**
**vs. male genital complexity**	**52.721**	**0.001**	**−1.936 (0.411)**	**0.001**
vs. female body length	1.511	0.487	7.272 (3.276)	0.056
vs. male body length	0.329	0.746	0.430 (0.575)	0.476
vs. sexual size dimorphism	0.087	0.893	0.372 (0.722)	0.619
**vs. sexual cannibalism rate**	**9.667**	**0.018**	**−0.277 (0.081)**	**0.025**

Significant associations are bolded

GLMM analyses (Additional file 5: file S2) establish a negative correlation between male mating rates and factor MR3 that combined male and female genital complexity. This implies that monogyny can be predicted by high genital complexity in both sexes. By not revealing a significant correlation between female mating rates and MR1-3, these analyses do not directly support our prediction about female mating rates and male genital complexity.

Consistent with our second prediction, both phylogenetic ANOVA and GEE analyses (Table 1) establish that male mating rates are negatively associated with rates of sexual cannibalism (with monogynous species being more cannibalistic).

## Discussion and conclusions

**Fig. 3:**
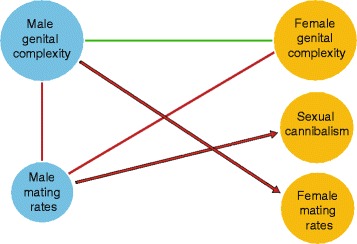
Summary relationships between studied phenotypes detected by different comparative analyses. Lines mark significant associations; *green* and *red lines* denote positive and negative associations, respectively. *Arrows* imply direction as derived from specific predictions

The results from our comparative analyses, summarized in Fig. 3, support our prediction that female mating rates are negatively associated with male genital complexity. We also found male mating rates to be negatively associated with male genital complexity. As predicted, sexual cannibalism is negatively correlated with male mating rates (Fig. 3). Interestingly, we found no association between female mating rates and female gigantism (or SSD), and thus the evolution of body size *per se* does not appear to be linked with mating systems.

Complex male genital organs, functioning as effective mating plugs to enforce monandry, have evolved from an ancestral polyandrous mating system (Fig. 2). Experimental studies reveal that these complex male genitals, when mutilated, effectively plug female copulatory openings [40, 41, 51] but subsequently limit male re-mating opportunities. A negative correlation between female mating rates and male genital complexity provides support for the idea that in relatively tiny nephilid spider males, observed increases in palpal complexity limit female remating opportunities, and thus the evolution of genital complexity promotes sexual conflict. We interpret these patterns to imply that complex male genitals act as a male persistence mechanism by enforcing monandry through effective genital plugging.

While the specific costs of reduced mating rates to plugged females are rarely documented, the general benefits of polyandry [20] suggest that male-enforced monandry in nephilids does not serve female interests [26]. Hence one would expect to detect female resistance mechanisms, either through morphological adjustments (genital complexity), or behavioral adaptation, e.g., sexual cannibalism. While the latter seems to coevolve with monogyny, the former is not directly supported by our analyses. To elaborate, our analyses confirmed the predicted negative association between sexual cannibalism and male mating rates, and additionally found that species with larger females have simpler genitals. We interpret these results to imply that post-copulatory sexual cannibalism acts as female resistance mechanism to male monopolization. However, female resistance traits should also reassert polyandry, but it does not seem that adjustments to female genital complexity function in this manner. Namely, the absence of a correlation between female genital complexity and female mating rates suggests that genital morphology modifications do not serve as female resistance mechanism [26].

These emerging patterns should be interpreted cautiously for several reasons. First, evolutionary processes that generate genital variation may not be detectable by correlated patterns alone [22]. In an antagonistic coevolutionary process, adaptations and counter adaptations are ongoing processes that counterbalance each other, and whose continuum blurs the imprint of sexual conflict [17]. Following this logic, it is the evolutionary outliers, i.e., adaptations of one sex departing from the continuum, that are informative of evolutionary processes [17]. We cannot claim with any certainty that the phenotypes comprising the present study represent such outliers. Nevertheless, integrative comparative analyses may inform evolutionary processes [87], and our study, which integrates the currently available behavioral, experimental and functional evidence (Additional file 1: Table S1) with phylogenetically controlled comparative analyses, supports at least a partial role of sexual conflict in spider phenotypic evolution.

A second caveat is that our study inferred mating rates, and simplified them into scores of monogamy versus polygamy. Ideally, mating rate data would include real variation on measured mating frequencies, but currently these data are largely unavailable for nephilid spiders, and would, in any case, likely differ between populations. The inferred female mating rates are based on our understanding of a morphological-behavioral outcome, i.e., single versus multiple mating plugs. This approach aligns with rates reported for taxa for which experimental data are available (Additional file 1: Table S1).

While several studies of insects and arachnids have detected coevolutionary patterns of reproductive traits between the sexes (e.g., fruit flies [88]; and harvestmen [89]), ours differs because it specifically links genital complexity with sex-specific mating rates (see also Rowe and Arnqvist [22] for water striders). The previously reported positive correlation between male and female genital complexity [26], is stronger with the new phylogeny (Fig. 1), which is topologically quite different from the prior hypothesis (Additional file 4: Figure S2) and implies that the evolutionary signal is robust.

Sexual conflict is not the only possible explanation for patterns of correlated evolution of genitalia found in several animal groups [7, 88, 90]. Such coevolutionary patterns could also result from the lock and key mechanism, male-male competition, or female choice, or a combination of them [91]. Thus, our discussion of the evidence in support of sexual conflict in spiders does not imply the absence of other mechanisms related to sexual selection.

For example, features of male palps may function to stimulate females, thereby introducing the possibility of cryptic female choice [92, 93]. However, the literature on the functional significance of male palpal hooks and processes (Additional file 3: File S1) suggests their function in grasping, mounting, and manipulating the female, and a role in genital mutilation and plugging. This functional morphological evidence, the detected phylogenetic correlations among phenotypes, and the lack of described behavioral and physiological stimulatory mechanisms, combined suggest that stimulation is an unlikely explanation for these male morphologies, but rather points towards monopolization of females via genital plugging.

## Additional files

Additional file 1: Table S1. Nephilid spider and outgroup data for all variables used in phylogenetic comparative analyses. Mating rates are inferred based on experimental and morphological evidence. Separate (Excel) file. (XLSX 16 kb)
Additional file 2: Figure S1. Relatively simple (left; *Clitaetra*) and complex (right, *Herennia*) genital morphology in nephilid spiders. Upper images show distal parts of the male pedipalp, lower images show female epigyna. Note that the male embolic conductor (EC) interacts with the copulatory opening (CO) of the female, and if broken off, may form an elaborate mating plug (lower right). (EPS 7104 kb)
Additional file 3: File S1. Morphological features contributing to scores of genital complexity and their hypothesized function. Separate (Word) file. (DOCX 16 kb)
Additional file 4: Figure S2. Contrasting phylogenetic topologies: A, cladogram from Kuntner et al. (2008) with no branch length information; B, Bayesian tree from Kuntner et al. (2013) with rearranged taxonomic relationships and branches proportional to evolutionary change. See Methods for additional detail. Separate (pdf) file. (PDF 290 kb)
Additional file 5: File S2. The code and the results of the GLMM analyses. Separate Word file. (DOCX 523 kb)
Additional file 6: Figure S3. Relationships of studied phenotypes with female and male inferred mating rates (raw, species data). Relationships that become significant after phylogenetic correction are highlighted. (PDF 25 kb)

## Abbreviations

FBL: Female body length (mm)
FGC: Female genital complexity
MBL: Male body length (mm)
MGC: Male genital complexity
SSD: Sexual size dimorphism
