# Supplementary material for: The evolution of genital complexity and mating rates in sexually size dimorphic spiders
Source: BMC Evol Biol. 2016 Nov 9;16:242. doi: 10.1186/s12862-016-0821-y (PMC5103378; doi:10.1186/s12862-016-0821-y)
Supplement: Additional file 3: File S1. — Morphological features contributing to scores of genital complexity and their hypothesized function. Separate (Word) file. (DOCX 16 kb) [file 12862_2016_821_MOESM3_ESM.docx]

The methodology is from Kuntner et al. (2009a), binary characters are less (0) versus more complex (1). Hypothesized function of each morphological feature (1-10 for males, 11-20 for females) is listed.

1 Embolic conductor membrane: (0) absent; (1) present.

A membrane is sometimes present on the basis of the embolic conductor in nephilids and araneids (Kuntner et al. 2008, 2009a: fig. 1). While its function is unclear in nephilids, membranes between sclerotized parts of the terminal parts of the palp add to maneuverability during mating as they expand and place/rotate palpal parts into position.

2 Embolic conductor shape: (0) finger-like; (1) broad.

The variation in nephilids is dichotomous (Kuntner et al. 2008, 2009a: fig. 1). Behavioral studies have demonstrated that broad embolic conductors make more effective genital plugs compared with thin ones (Kuntner et al. 2009b; Kralj-Fišer & Kuntner 2012) as they close the female genital opening more thoroughly.

3 Embolic conductor (division): (0) entire; (1) subdivided into two or more sclerites.

In araneids, but not nephilids, the embolic conductor is divided into a proximal and a distal sclerite that imply functional differences (Kuntner et al. 2008, 2009a: fig. 1).

4 Distal embolic conductor flap: (0) absent; (1) present.

Only some *Herennia* species possess such modification of the distal part of the embolic conductor (Kuntner et al. 2008, 2009a: fig. 1). Its function has not been tested, but it may reasonably be assumed to enlarge the space, and thus effectiveness, of embolic plug, a seemingly obligate *Herennia* behavior (Kuntner et al. 2009b).

5 Embolic conductor edge: (0) smooth; (1) ridged.

Since the ridges on the distal part of the embolic conductor (Kuntner et al. 2008, 2009a: fig. 1) are only found in those nephilids that plug, we assume that their function may be to secure more friction compared with smooth edges.

6 Embolic conductor curvature: (0) more/less straight; (1) sigmoidal/bent distally.

Simple embolic conductors are linear, but more complex ones exhibit curvatures and bends (Kuntner et al. 2008, 2009a: fig. 1) that may logically make decoupling more difficult.

7 Embolic conductor tip: (0) straight; (1) hooked.

A hook present at the terminal end of the embolic conductor in *Nephila fenestrata* (Kuntner et al. 2008, 2009a: fig. 1) has been hypothesized to anchor the palp in place during mating, and to facilitate breakage and plugging (Fromhage & Schneider 2006).

8 Embolic conductor subdistal protuberance: (0) absent; (1) present.

A protuberance near the terminal end of the embolic conductor in *Nephila plumipes* and *Nephila fenestrata* (Kuntner et al. 2008, 2009a: fig. 1) may add to successful anchoring of the palp that facilitates embolic breakage.

9 Embolus relative length: (0) short to medium (<2x cymbium length); (1) long (>2x cymbium length).

Some nephilids exhibit exaggerated lengths of their emboli (the penetrating, sperm-transferring sclerite) (Kuntner et al. 2008, 2009a: fig. 1). These needle like, but flexible structures can penetrate curved female copulatory ducts and reach into spermathecae. They can bypass existing plugs (Kuntner et al. 2009b).

10 Embolus: (0) smoothly curved; (1) sharply hooked.

A nephilid/araneid embolus may sport a hook (Kuntner et al. 2008) that likely prevents withdrawal, thereby facilitating embolic breakage in the female duct.

11 Epigynal openings: (0) superficial; (1) in chambers.

A female copulatory opening may lie in an enlarged chamber (Kuntner et al. 2008, 2009a: fig. 2) that adds volume to the space needed to be filled by an embolic plug.

12 Epigynal septum: (0) absent; (1) present.

An integumental divide between copulatory openings may be simple or it may be shaped, the latter called septum (Kuntner et al. 2008, 2009a: fig. 2) whose function is obscure.

13 Epigynal paired sclerotized pocket: (0) absent; (1) present.

A lateral sclerotization may surround the chambers (Kuntner et al. 2008, 2009a: fig. 2).

14 Epigynal scape: (0) absent; (1) present.

An araneid scape serves specific male sclerites during mating (Mouginot et al. 2015), in our taxonomic sample it is only relevant to *Nephila senegalensis* (Kuntner et al. 2008, 2009a: fig. 2).

15 Paired epigynal flap: (0) absent; (1) present.

In the araneid *Deliochus*, epigynal flaps (Kuntner et al. 2008, 2009a: fig. 2) add to the epigynal complexity but serve an unknown function.

16 Copulatory opening form: (0) elongated slit openings; (1) rounded openings.

Rounded openings provide more space for male sclerites (Kuntner et al. 2008, 2009a: fig. 2).

17 Copulatory duct morphology: (0) short (wider than long); (1) long (longer than wide).

The length of copulatory ducts (Kuntner et al. 2008, 2009a: fig. 2) affects the feasibility and speed at which males can reach spermathecae.

18 Spermathecae: (0) unlobed; (1) lobed.

Lobed spermathecae (Kuntner et al. 2008, 2009a: fig. 2) provide additional voluminous structures for sperm storage.

19 Epigynal sclerotized arch: (0) absent; (1) present.

An additional sclerotization within the epigynum (Kuntner et al. 2008, 2009a: fig. 2) may provide structural support for heavily sclerotized spermathecae.

20 Copulatory duct curvature: (0) none or slight; (1) curved or looped.

Duct curves (Kuntner et al. 2008, 2009a: fig. 2) may affect the feasibility and speed at which males can reach spermathecae.
