# Supplementary figures and images for: The evolution of genital complexity and mating rates in sexually size dimorphic spiders
Source: BMC Evol Biol. 2016 Nov 9;16:242. doi: 10.1186/s12862-016-0821-y (PMC5103378; doi:10.1186/s12862-016-0821-y)

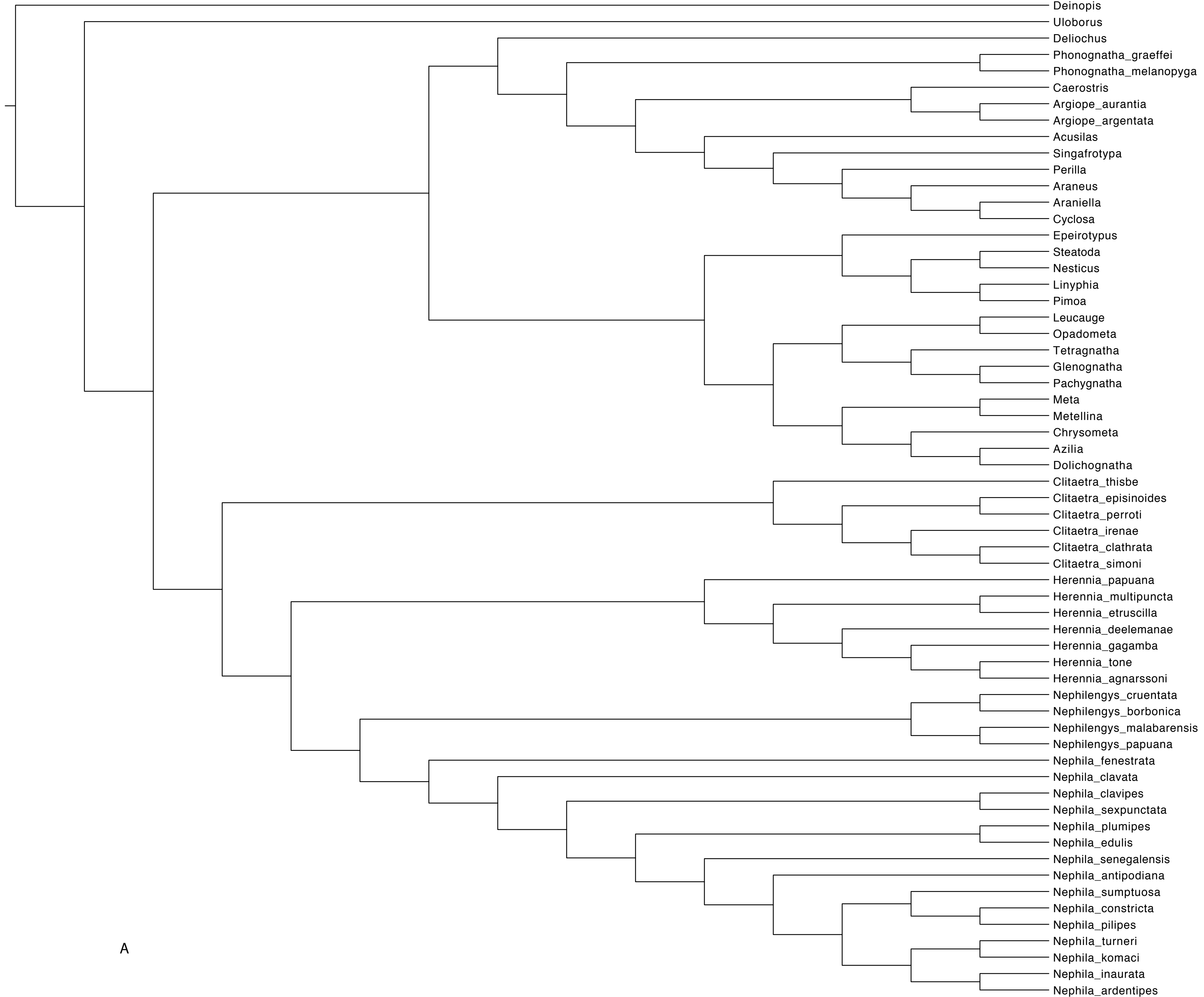

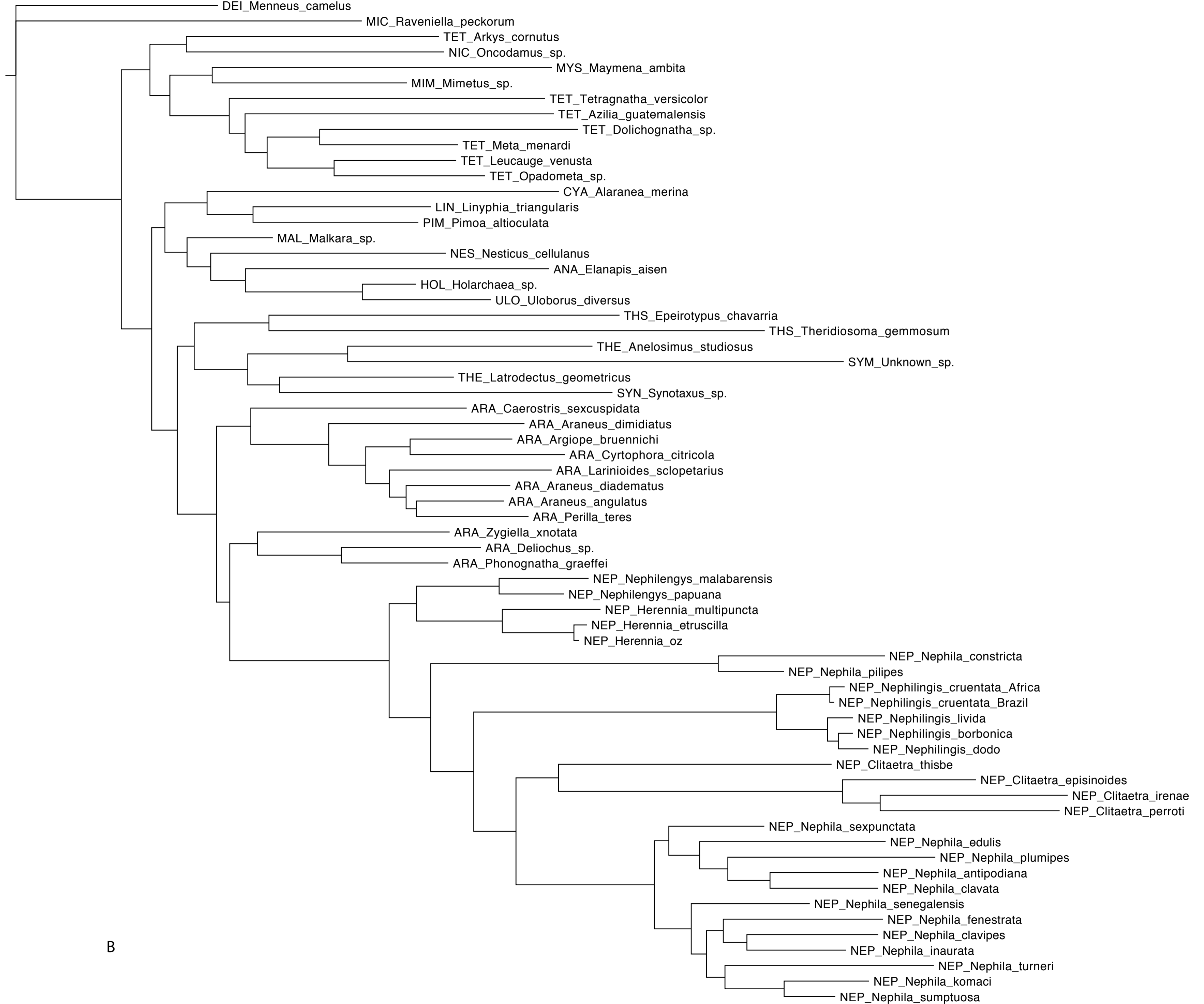

B

0.1

Supplement: Additional file 4: Figure S2. — Contrasting phylogenetic topologies: A, cladogram from Kuntner et al. (2008) with no branch length information; B, Bayesian tree from Kuntner et al. (2013) with rearranged taxonomic relationships and branches proportional to evolutionary change. See Methods for additional detail. Separate (pdf) file. (PDF 290 kb) [file 12862_2016_821_MOESM4_ESM.pdf]

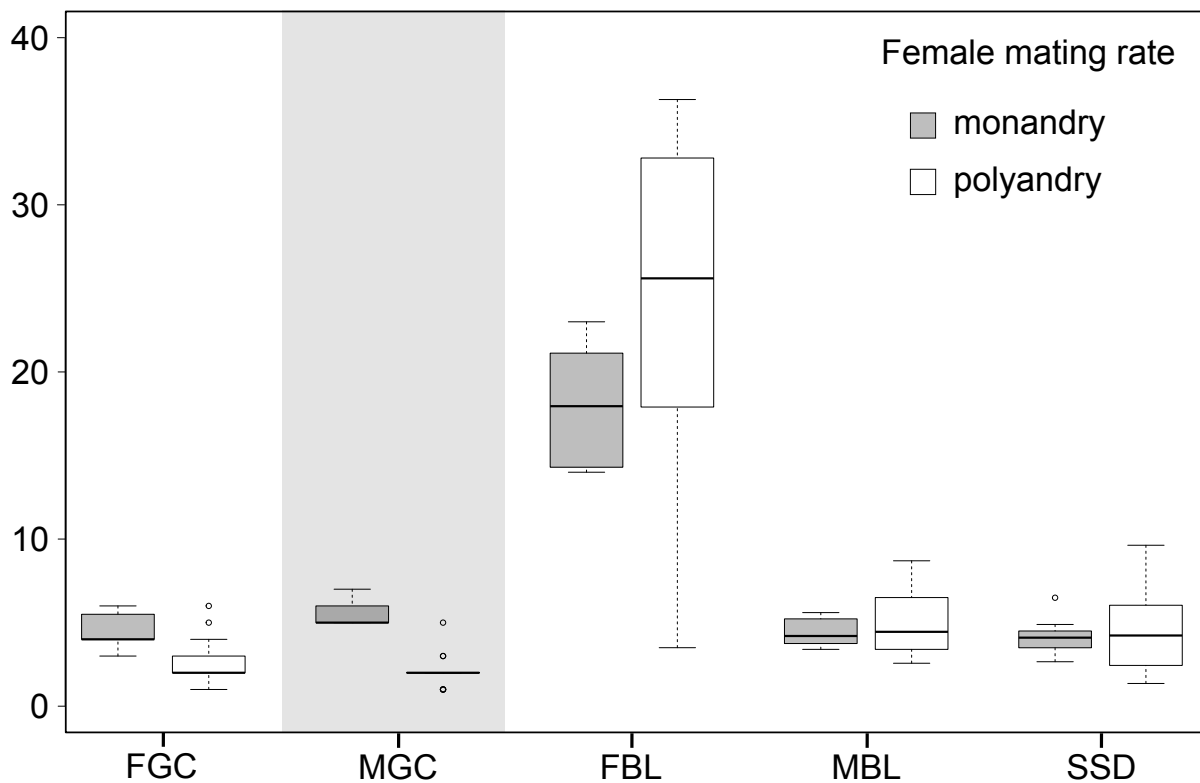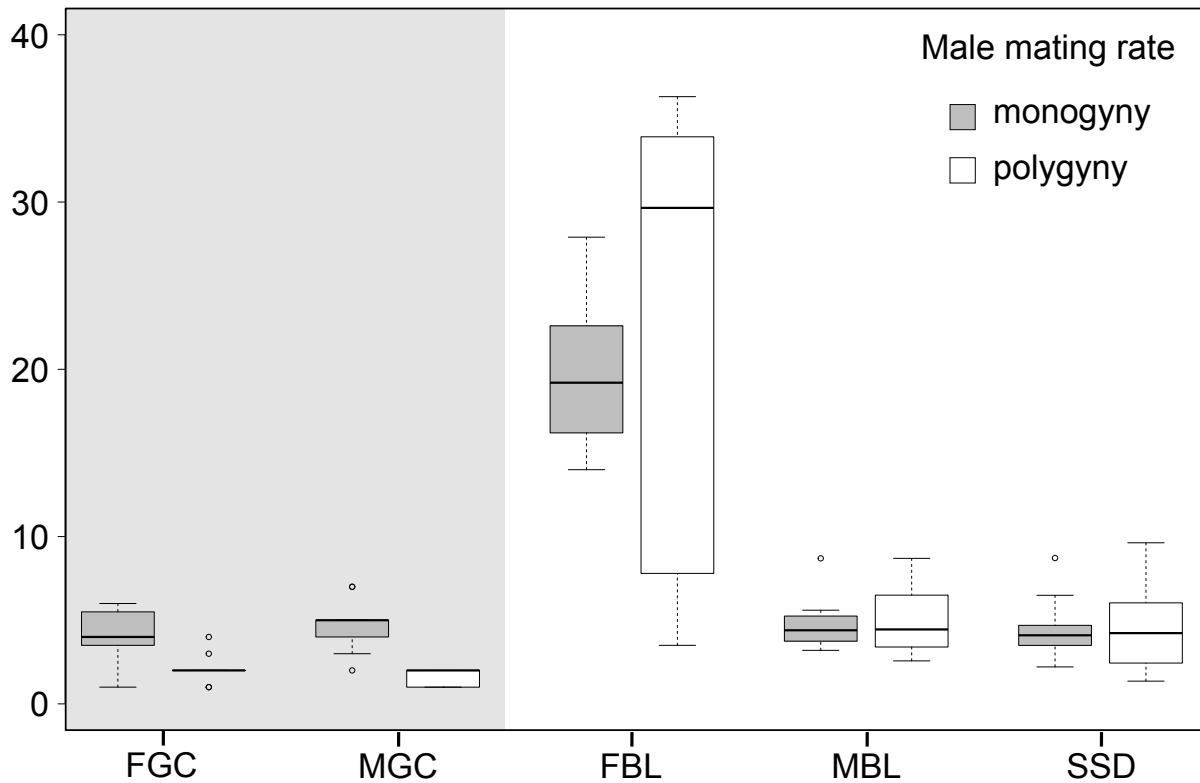

Supplement: Additional file 6: Figure S3. — Relationships of studied phenotypes with female and male inferred mating rates (raw, species data). Relationships that become significant after phylogenetic correction are highlighted. (PDF 25 kb) [file 12862_2016_821_MOESM6_ESM.pdf]
