# Supplementary material for: The evolution of genital complexity and mating rates in sexually size dimorphic spiders
Source: BMC Evol Biol. 2016 Nov 9;16:242. doi: 10.1186/s12862-016-0821-y (PMC5103378; doi:10.1186/s12862-016-0821-y)
Supplement: Additional file 5: File S2. — The code and the results of the GLMM analyses. Separate Word file. (DOCX 523 kb) [file 12862_2016_821_MOESM5_ESM.docx]

Factor analysis

> library(psych)

> fit <- fa(dat, nfactors=3, rotate="oblimin")

> fa.sort(fit)

Factor Analysis using method = minres

Call: fa(r = dat, nfactors = 3, rotate = "oblimin")

Standardized loadings (pattern matrix) based upon correlation matrix

MR1 MR3 MR2 h2 u2 com

SSD 1.00 0.03 -0.23 0.99 0.0066 1.1

FBL 0.82 -0.09 0.44 0.99 0.0072 1.6

MGC 0.02 0.84 0.10 0.66 0.3357 1.0

FGC 0.00 0.79 -0.06 0.65 0.3485 1.0

MBL -0.04 -0.01 0.98 0.96 0.0437 1.0

MR1 MR3 MR2

SS loadings 1.68 1.34 1.23

Proportion Var 0.34 0.27 0.25

Cumulative Var 0.34 0.61 0.85

Proportion Explained 0.40 0.32 0.29

Cumulative Proportion 0.40 0.71 1.00

With factor correlations of

MR1 MR3 MR2

MR1 1.00 -0.17 0.10

MR3 -0.17 1.00 -0.25

MR2 0.10 -0.25 1.00

Mean item complexity = 1.1

Test of the hypothesis that 3 factors are sufficient.

> fa.diagram(fit, simple=F, side=4, cex=12/12, cut=.1)


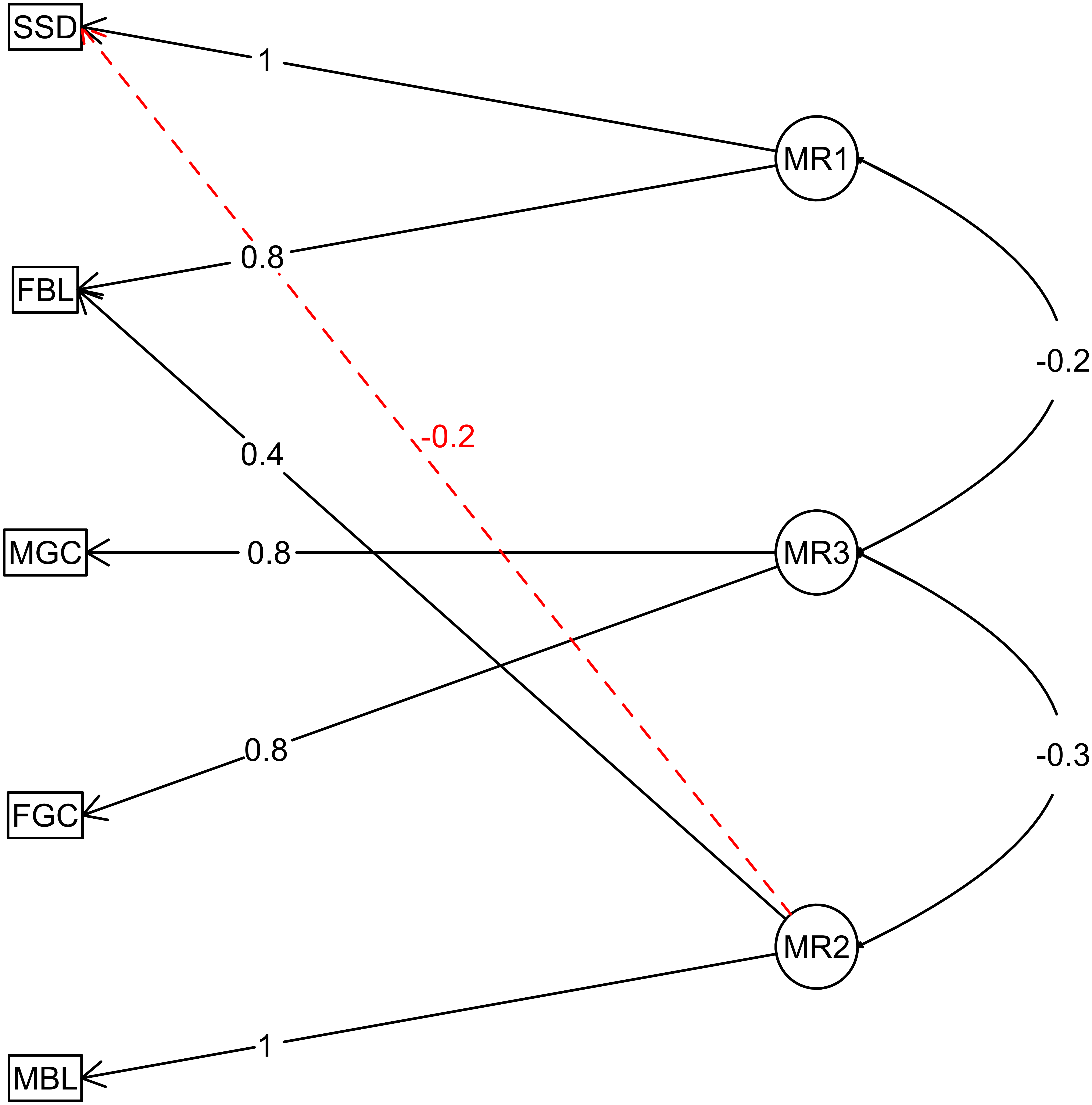


MMR

> phylo <- read.nexus("chrotree.tre")

> data <- read.table("MCMCglmm_RD.txt",header=T)

> inv.phylo <- inverseA(phylo,nodes="TIPS",scale=TRUE)

> prior <- list(

B=list(mu = rep(0,4), V = diag(4) * (10 + pi^2/3)),

G=list(G1=list(V=1,nu=0.002)),

R=list(V=1,nu=0.002))

> model_MMR <- MCMCglmm(

MMR~MR1+MR2+MR3, random=~phylo,

family="categorical", ginverse=list(phylo=inv.phylo$Ainv),

prior=prior,data=data,nitt=1e6,burnin=2.5e5,thin=200)

> summary(model_MMR)

Iterations = 250001:999801

Thinning interval = 200

Sample size = 3750

DIC: 9.839649

G-structure: ~phylo

post.mean l-95% CI u-95% CI eff.samp

phylo 4.697 0.0002298 25.05 164.7

R-structure: ~units

post.mean l-95% CI u-95% CI eff.samp

units 1.367 0.0001891 6.762 747

Location effects: MMR ~ MR1 + MR2 + MR3

post.mean l-95% CI u-95% CI eff.samp pMCMC

(Intercept) 0.2397 -2.4732 3.0297 651.7 0.868267

MR1 0.1826 -2.2942 2.7376 344.1 0.852267

MR2 -0.6209 -2.8660 1.6576 384.3 0.577067

MR3 -7.3911 -11.2077 -3.4144 198.4 0.000533 ***

---

Signif. codes: 0 ‘***’ 0.001 ‘**’ 0.01 ‘*’ 0.05 ‘.’ 0.1 ‘ ’ 1

FMR

> Prior1 <- list(

B=list(mu = rep(0,4), V = diag(4) * (10 + pi^2/3)+1),

G=list(G1=list(V=10,nu=0.002)),

R=list(V=10,nu=0.002))

> model_FMR <- MCMCglmm(

FMR~MR1+MR2+MR3, random=~phylo,

family="categorical", ginverse=list(phylo=inv.phylo$Ainv),

prior=prior1,data=data,nitt=1e7,burnin=2.5e5,thin=200)

> summary(model_FMR)

Iterations = 2500001:9999001

Thinning interval = 1000

Sample size = 7500

DIC: 2.21854

G-structure: ~phylo

post.mean l-95% CI u-95% CI eff.samp

phylo 6182 0.003201 27610 14.14

R-structure: ~units

post.mean l-95% CI u-95% CI eff.samp

units 112.5 0.002023 445 495.5

Location effects: FMR ~ MR1 + MR2 + MR3

post.mean l-95% CI u-95% CI eff.samp pMCMC

(Intercept) 0.5607 -6.4054 7.3724 2668.6 0.853

MR1 0.2023 -6.3437 6.7046 3106.2 0.945

MR2 -0.2047 -6.3626 6.2016 2578.7 0.932

MR3 -2.0984 -9.0789 5.0080 309.2 0.543
